# A robust web-based tool to predict viral shedding in patients with Omicron SARS-CoV-2 variants

**DOI:** 10.1183/23120541.00939-2023

**Published:** 2024-05-20

**Authors:** Weilong Zhang, Xiaoyan Gai, Ben Wang, Zhonghui Duan, Qingtao Zhou, Lili Dai, Changjian Yan, Chaoling Wu, Jiarun Fan, Ping Wang, Ping Yang, Fang Bao, Hongmei Jing, Chao Cai, Chunli Song, Yingmin Ma, Yongchang Sun

**Affiliations:** 1 Department of Hematology, Lymphoma Research Center, Peking University Third Hospital, Beijing, China; 2 Department of Respiratory and Critical Care Medicine, Peking University Third Hospital, and Center for Chronic Airway Diseases, Peking University Health Science Center, Peking University, Beijing, China; 3 Orthopedics Department, Peking University Third Hospital, Beijing, China; 4 Department of Respiratory and Critical Care Medicine, Beijing Youan Hospital, Capital Medical University, Beijing, China; 5 W. Zhang, X. Gai and B. Wang contributed equally to this article as co-first authors; 6 Y. Ma and Y. Sun contributed equally to this article as lead authors and supervised the work

## Abstract

**Background:**

Data on viral kinetics and variants affecting the duration of viral shedding were limited. Our objective was to determine viral shedding in distinct severe acute respiratory syndrome coronavirus 2 variants, including Omicron BA.4/5 and BF.7, and to identify the relevant influencing factors.

**Methods:**

We carried out a longitudinal cohort study at Beijing Xiaotangshan Fangcang shelter hospital from May to June 2022 (Omicron BA.4/5) and from November to December 2022 (Omicron BF.7). Nucleocapsid protein (N) and open reading frame (ORF) genes were considered as the target genes of the reverse transcription PCR. The daily results of cycle threshold (CT), including lowest ORF1ab-CT values for days 1–3 post-hospitalisation and lowest N-CT values for days 1–3 post-hospitalisation (CT3minN) and demographic and clinical characteristics were collected.

**Results:**

1433 patients with coronavirus disease 2019 (COVID-19) were recruited from the Fangcang shelter hospital, in which 278 patients were diagnosed with Omicron BA.4/5 and 1155 patients with Omicron BF.7. Patients with BF.7 infection showed a longer duration of viral shedding. The duration of viral shedding was associated with the variants age, alcohol use, the severity of COVID-19 and CT3minN. Moreover, the nomogram had excellent accuracy in predicting viral shedding.

**Conclusions:**

Our results indicated that patients with Omicron BF.7 had a longer period of contagiousness than those with BA.4/5. The duration of viral shedding was affected by a variety of factors and the nomogram may become an applicable clinical instrument to predict viral shedding. Furthermore, we developed a new COVID-19 viral shedding predicting model that can accurately predict the duration of viral shedding for COVID-19, and created a user-friendly website to apply this prediction model (https://puh3.shinyapps.io/CVSP_Model/).

## Shareable abstract

**A web-based tool to predict viral shedding in patients with Omicron variants of SARS-CoV-2** https://bit.ly/48sfyCP

## Introduction

Coronavirus disease 2019 (COVID-19) is currently circulating around the world. The Omicron variant, first confirmed in South Africa on 11 November 2021, has become the primary variant of COVID-19 in the global pandemic [1]. In accordance with Centers for Disease Control and Prevention (CDC) guidelines, it has been recommended that the isolation duration for individuals with COVID-19 be shortened from 10 days after the onset of symptoms or the positive result of a nucleic acid test to 5 days, followed by wearing a mask for 5 days [2]. What is noteworthy is that the Omicron variant is potentially three times more infectious than the Delta variant, with a shorter duration of incubation, but with a lower viral load at the moment of diagnosis than the previously prevalent lineages of severe acute respiratory syndrome coronavirus 2 (SARS-CoV-2) [3–5]. According to national genetic sequencing tests, the recently prevalent strains are the Omicron variants BF.7 (short for BA.5.2.1.7) and BA.4/5; they are highly contagious viruses, but with a low mortality rate after infection [6, 7]. Unfortunately, viral kinetics and the duration of viral shedding of the two Omicron variants, as well as the associated affecting factors, have remained unclear until now.

The factors that influence viral dynamics of SARS-CoV-2 [8] include age [9, 10], gender [11], comorbidities [12], delayed hospitalisation/treatment [11–13], disease severity and immunity [10, 11, 14–17]. Several studies have shown that gender and age affect viral kinetics [18, 19]. According to a study from China, viral RNA was detectable in male patients infected with SARS-CoV-2 for a longer time compared to female patients [16]. What is more, in cases of SARS-CoV-2 infection, viral RNA was cleared more rapidly in individuals aged <18 years, but more slowly in those aged ≥50 years [20], implying that the younger the age, the shorter the viral shedding. A possible association between gender and viral kinetics was controversial, because other studies have suggested that female patients with COVID-19 have longer viral shedding [21]. In addition, there were conflicting data regarding the timing of viral shedding in asymptomatic and symptomatic patients with COVID-19. A study on SARS-CoV-2 showed that viral RNA was cleared faster in asymptomatic subjects than in symptomatic ones [22], whereas the duration of viral RNA shedding in symptomatic subjects was shorter according to Long *et al*. [23]. Furthermore, the study observed that moderate to severe immunocompromise was associated with higher nasal viral loads at the time of enrolment, and immunosuppression was found to result in increased viral shedding and altered decay kinetics of SARS-CoV-2 [17].

The duration of viral shedding is a major marker for the elimination of transmission risk in patients with COVID-19 [24]. However, as there was considerable variability among the various studies, further exploration of relevant factors in large samples was needed. Thus, we conducted this study in order to further investigate the relevant factors affecting viral shedding and the relationship between viral shedding and clinical characteristics, and to construct a nomogram to predict the duration of viral shedding.

## Methods and materials

### Participant recruitment and data collection

This study was performed in the Beijing Xiaotangshan Fangcang shelter hospital (Beijing, China), in accordance with the guidelines of the Declaration of Helsinki, and was approved by the ethics committee of Peking University Third Hospital. The participants were recruited from May to June 2022 (BA.4/5), and from October to November 2022 (BF.7) in the shelter hospital. The identification of Omicron BA.4/5 and BF.7 variants was aligned with reports of the Chinese Center for Disease Prevention and Control and Global Initiative on Sharing All Influenza Data during the two-epidemic period. 1433 patients were recruited for the present study. Inclusion criteria were as follows. The sample of nasopharyngeal swabs of each patient was detected by the testing centre of the Fangcang shelter hospital and the positive result of COVID-19 was confirmed by nucleic acid test. Only patients with asymptomatic infection (confirmed COVID-19, but no clinical symptoms) and with mild disease (mild symptoms, but no pneumonia on chest imaging) were included. There was no age restriction for enrolment. Exclusion criteria applied if the patient was in a critical condition and was unable to complete nucleic acid test, or was transferred out of the Fangcang shelter hospital in the process of the study. Demography and clinical characteristics were collected from electronic medical records. The severity of COVID-19 was divided into two types: “asymptomatic infection” and “mild type” [25].

### Measurement of viral load using quantitative PCR

Nucleic acid testing was performed in a continuous manner (seven times on average for each patient). Nucleocapsid (N) and open reading frame lab (ORFlab) were considered as the target genes of quantitative real-time PCR (qPCR) [26]. The extraction and detection of viral RNA from the sample of the swabs were performed in accordance with the manufacturer's instructions using the Marburgvirus Real Time PCR Kit (Shanghai Biogerm Medical Technology, Shanghai, China). Viral load as determined by qPCR was expressed as cycle threshold (CT) value [27]. The viral shedding cut-off threshold of CT was set as 35. If the CT value of both N and ORFlab was >35, it was considered to be a negative result; otherwise, a positive result. If the CT value of the patient was >35, the nucleic acid test was performed the following day to confirm the negative result; otherwise, the daily nucleic acid test was performed until the CT value was >35, and the negative result was confirmed the following day. The duration of viral shedding was identified as the interval between the day of the first negative result and the day of diagnosis or the onset of symptoms [11, 14].

#### Technical replicates

Our routine qPCR testing methodology involved performing technical replicates to ensure the reliability of the results. For each sample, the qPCR assay was typically conducted in triplicate to minimise experimental variability.

#### Limit of detection or sensitivity

The limit of detection and sensitivity of the qPCR assay was 200 copies·mL^−1^. While the specific limit of detection could vary based on factors such as the RNA extraction method and assay design, our testing protocol adhered to recommended guidelines, ensuring the reliable detection of SARS-CoV-2.

#### Standard curves for ORF1ab and N targets

There were no standard curves for ORF1ab and N targets, as this was not a quantitative test. Our qPCR included three negative controls and one positive control to ensure the reliability of the results.

### Establishment of a viral shedding prediction model

We incorporated all variables into the modelling process. The stepAIC function from the MASS package was utilised for bidirectional stepwise regression model selection, ultimately resulting in the optimal prediction model. The predicted values of viral shedding were calculated as 15.58−0.31 × CT3minN+1.01 × diagnosis−0.73277 × alcohol use+0.68 × variant (where CT3minN represents the lowest N-CT values for days 1–3 post-hospitalisation). Additionally, the area under the curve (AUC) value was computed. The data were then divided into a training set and a validation set in an 8:2 ratio, with the AUC values calculated separately for each.

### Visualisation of nomogram

The results of the CVSP model were visualised using a nomogram. The nomogram assigned a score to each value level of the indicators based on the contribution of variant, alcohol use, diagnostic type and CT3minN to the outcome variable, which was represented by the size of the regression coefficient. For categorical variables, represented by squares, a larger square area indicated a larger sample size for the respective category. For continuous variables, the density curve displayed the data distribution. A total score could be obtained by summarising scores of multiple predictors for each patient. The functional conversion relations between total scores and outcome variables could then be used to calculate the predictive value of viral shedding for each patient (supplementary figure S2B).

### Statistical analysis

All statistical analyses were performed in R (version 4.0) and the normality of the data was tested by the Kolmogorov–Smirnov test. Continuous variables were presented as mean±sd and categorical variables were presented as n (%). Continuous variables were tested using the t-test or one-way ANOVA when appropriate. Categorical variables were tested using the Chi-squared test or Fisher's exact test when appropriate. Correlation coefficient and prediction capability were assessed by Spearman correlation analysis and receiver operating characteristic (ROC) analysis, respectively. The “ggplot2” and “Regplot” R packages were used to draw figures. p<0.05 was considered statistically significant.

## Results

### Baseline characteristics of patients with Omicron BA.4/5 or BF.7

1433 patients were enrolled in the present study, of whom 278 were diagnosed with Omicron BA.4/5 from May to June 2022 (supplementary table S1). The remaining 1155 patients were diagnosed with Omicron BF.7 from November to December of 2022. Although the proportions of the sexes were similar, the mean age of the BF.7 group was higher than the BA.4/5 group. The severity of COVID-19 was comparable between BA.4/5 and BF.7 groups and the majority of patients showed no fever. The proportion of alcohol use, smoking, vaccination and blood neutrophil count in the BA.4/5 group was higher relative to the BF.7 group. Conversely, the proportion of patients complicated with hypertension along with diabetes mellitus was moderately higher in the BF.7 group. Levels of inflammatory markers, including C-reactive protein, blood lymphocytes, white blood cells and platelets, were similar between the BA.4/5 group and the BF.7 group. Moreover, viral shedding of the BF.7 group was shorter compared with the BA.4/5 group. The lowest ORF1ab-CT values for days 1–3 (CT3minORF) and CT3minN of the BF.7 group were significantly higher compared to the BA.4/5 group.

### Comparison between slow viral shedding and rapid viral shedding groups

According to the median of viral shedding, 721 patients were allocated into the slow viral shedding (SVS) group (12.423±1.524) and 712 patients into the rapid viral shedding (RVS) group (8.403±1.805). Subsequently, we performed a baseline comparison between SVS and RVS groups and several characteristics, which were markedly different between the two groups (supplementary table S2). The proportions of patients with BA.4/5 infection and patients with BF.7 infection were different between the two groups; the RVS group had more patients infected with BA.4/5 compared to the SVS group. There was no significant difference in the proportions of the sexes, but the mean age of the RVS group was younger than that of the SVS group. Asymptomatic infection accounted for a greater proportion of the RVS group compared to the SVS group. Moreover, CT3minORF and CT3minN were higher in the RVS group. The number of patients with diabetes mellitus in the SVS group was greater than in the RVS group. In addition, a series of clinical characteristics, including alcohol use, smoking, hypertension, fever, vaccination and inflammatory markers were similar between the two groups.

### Relationship of viral shedding with multiple clinical characteristics

**FIGURE 1:**
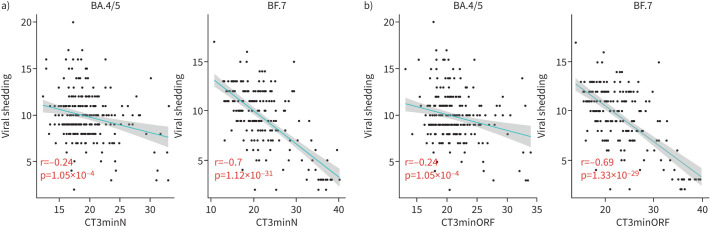
Correlation analysis of viral shedding with a) lowest nucleocapsid protein and cycle threshold (CT) values for days 1–3 post-hospitalisation (CT3minN) and b) lowest open reading frame 1ab and CT values for days 1–3 (CT3minORF).

**FIGURE 2:**
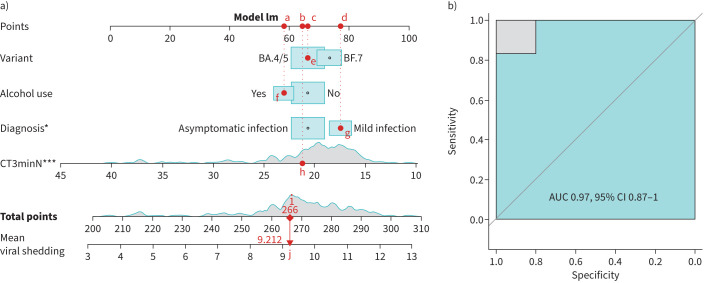
Establishment of the nomogram for predicting viral shedding of patients with BA.4/5 or BF.7. a) Nomogram with group, alcohol use, severity of coronavirus disease 2019, and lowest nucleocapsid protein and cycle threshold values for days 1–3 post-hospitalisation (CT3minN) for predicting viral shedding among patients. This figure presents a demonstrative example of the website we developed. If a patient is infected with the variant BA.4/5, it corresponds to point “e”, and its value is “c”. If the patient consumes alcohol, then “alcohol use” is “yes”, corresponding to point “f”, and its value is “a”. If the patient is diagnosed with a mild infection, then “diagnosis” is “mild infection”, corresponding to point “g”, and its value is “d”. The patient's “CT3minN” corresponds to point “h”, and its value is “b”. The sum of “a+b+c+d+h” equals “i”. Ultimately, a predicted viral shedding value is obtained, which is “j”. b) The receiver operating characteristic curve shows the accuracy of viral shedding prediction on the basis of the nomogram. AUC: area under the curve. *: p<0.05; ***: p<0.001.

Considering the difference of viral shedding between Omicron BA.4/5 and BF.7 (supplementary figure S1A), we further analysed the relationship between baseline characteristics and viral shedding. Patients with mild infection had significantly longer viral shedding than those with asymptomatic infection both in BA.4/5 and BF.7 groups (supplementary figure S1B). There was no difference in viral shedding between female and male patients in either the BA.4/5 or the BA.7 group (supplementary figure S1C). Interestingly, viral shedding was positively correlated with age, and the correlation coefficient was higher in BF.7 than in BA.4/5 (supplementary figure S1D). Patients who smoked and drank alcohol exhibited shorter viral shedding compared to those who did not in the BA.4/5 group (supplementary figure S1E,F). In addition, viral shedding was negatively correlated with both CT3minN and CT3minORF, and the correlation coefficient was higher in BF.7 than in BA.4/5 (figure 1a and b). To create an applicable clinical evaluation instrument to predict viral shedding among patients, we constructed a nomogram, including variants, alcohol use, severity of COVID-19 and CT3minN to predict viral shedding (figure 2a). ROC analysis indicated that the nomogram demonstrated excellent accuracy in predicting viral shedding across all samples, with an AUC of 0.97 (95% CI 0.87–1) (figure 2b). This high level of accuracy was consistent in both the 80% training set and the 20% validation set, with respective AUC values of 0.97 (95% CI 0.87–1) and 0.88 (95% CI 0.59–1) (supplementary figure S3A,B). A user-friendly website was created for facilitating clinicians to use our prediction model (https://puh3.shinyapps.io/CVSP_Model/). The input data (CT3minN, alcohol use, group and diagnosis) were pre-processed and then the predicted viral shedding duration was automatically output.

## Discussion

This study compared the viral shedding of patients infected with two strains of SARS-CoV-2, Omicron BF.7 *versus* Omicron BA.4/5, which has not been reported previously, and identified factors associated with viral shedding. Our study was a large sample study with 1433 patients infected with Omicron BF.7 or Omicron BA.4/5. In viral infections, the duration of viral shedding has been reported to be associated with infectivity and transmissibility and is a crucial factor in infection prevention and control [24]. Importantly, predicting viral shedding has important implications for communities, such as linking viral load data in sewage to the number of infected cases in the community to predict trends in clinically reported cases, which may be much higher than the number of reported cases [28–30]. As a result, it is vital to explore the viral shedding and its associated factors for individuals with Omicron variants [31].

Our research findings showed that the mean age of patients infected with Omicron BF.7 was older than that of patients infected with Omicron BA.4/5, suggesting that the two strains might not have the same susceptible population. In addition, subjects infected with Omicron BF.7 had significantly longer viral shedding compared to those infected with Omicron BA.4/5, implying that the BF.7 variant was more infectious and had higher transmission than the BA.4/5 variant. This might be attributed to the differences in SARS-CoV-2 stains [32]. Another reason might be that the proportion of patients complicated with comorbidities, such as hypertension and diabetes mellitus, was moderately higher in the BF.7 group in comparison to the BA.4/5 group, consistent with the results of Fu *et al*. [12], pointing out that patients with comorbidities experienced longer viral shedding.

Furthermore, it is worth noting that age, alcohol use, the severity of COVID-19 and CT3minN were associated factors affecting the duration of viral shedding. Viral shedding in the population of patients infected with BF.7 prolonged with age in an age-dependent manner and mean age of the RVS group was significantly younger than that of the SVS group, suggesting that younger people possibly have a faster ability to clear the virus than the general population, which was in keeping with previous studies [19, 33, 34]. One of the causes may have been immunosenescence [35]. Another cause was that the elderly had a higher content of angiotensin-converting enzyme 2 in the alveoli, which was deemed to be the receptor of the SARS-CoV-2 [36]. It is well known that older age could be a potentially essential predictive factor of severity and mortality for patients with COVID-19 [14, 37]. In both patients infected with BF.7 and those infected with BA.4/5, viral shedding was significantly shorter in patients with asymptomatic infection than in mildly infected patients, similar to the results of Kissler *et al*. [22], but showed an opposite trend to the results of Long *et al*. [23]. This phenomenon was probably due to the variability of the distinct SARS-CoV-2 strains. Moreover, viral shedding was negatively correlated with CT values, which was consistent with the research of Zou *et al*. [38], who reported that the severity of COVID-19 was possibly related to lower CT values. A nomogram, including variants, alcohol use, severity of COVID-19 and CT3minN, was established to create an applicable clinical evaluation instrument to predict viral shedding among patients, and ROC analysis revealed the excellent accuracy of the nomogram in predicting viral shedding.

Our investigation had several limitations. First, as a designated hospital for COVID-19, the majority of patients admitted to the Beijing Xiaotangshan Fangcang shelter hospital were asymptomatic, or had mild disease. Therefore, more severe and critically ill cases were not included in this study, which may lead to sampling bias. Second, the time of exposure could not be clearly determined in asymptomatic participants, limiting the interpretations of changes in viral shedding over time for this group.

### Conclusion

By evaluating the viral kinetics of the two novel Omicron variants, BF.7 and BA.4/5, and the relevant factors affecting viral dynamics and viral shedding, our study has important implications for guiding public health practice and may help improve clinical management of patients with COVID-19. Furthermore, we have created a user-friendly website (https://puh3.shinyapps.io/CVSP_model/), which can be used with our model to precisely predict the duration of viral shedding.

## Supplementary material

10.1183/23120541.00939-2023.Supp1 **Please note:** supplementary material is not edited by the Editorial Office, and is uploaded as it has been supplied by the author.
Supplementary methods 00939-2023.SUPPLEMENT
Figure captions 00939-2023.SUPPLEMENT2
Supplementary figures 00939-2023.SUPPLEMENT3
