# Supplementary material for: A robust web-based tool to predict viral shedding in patients with Omicron SARS-CoV-2 variants
Source: ERJ Open Res. 2024 May 20;10(3):00939-2023. doi: 10.1183/23120541.00939-2023 (PMC11111115; doi:10.1183/23120541.00939-2023)
Supplement: Supplementary file 1 [file 00939-2023.SUPPLEMENT.pdf]

Figure Legends

Supplementary Figure 1. Relationship of VS with baseline characteristics.

(A) Comparison of VS between patients with BA.4/5 and patients with BF.7.

(B) Comparison of VS between patients with asymptomatic infection and patients with mild infection.

(C) Comparison of VS between female patients and male patients.

(D) Correlation analysis between VS and age.

(E) Comparison of VS between patients without smoke and patients with smoke.

(F) Comparison of VS between patients without alcohol use and patients with alcohol use.

Supplementary Figure 2

(A)Patient recruitment flowchart.

(B) Detailed flowchart of the nomogram.

Supplementary Figure 3

(A) ROC curve showed the accuracy of VS prediction in train set.

(B) ROC curve showed the accuracy of VS prediction in validation set.
