# Supplementary material for: A robust web-based tool to predict viral shedding in patients with Omicron SARS-CoV-2 variants
Source: ERJ Open Res. 2024 May 20;10(3):00939-2023. doi: 10.1183/23120541.00939-2023 (PMC11111115; doi:10.1183/23120541.00939-2023)
Supplement: Supplementary file 2 [file 00939-2023.SUPPLEMENT2.pdf]

Supplementary Materials

Supplementary Table 1. Baseline characteristic of patients with Omicron BA.4/5 or BF.7.

Characteristic	level	Overall	BA.4/5	BF.7	p
n		1433	278	1155	
VS group (%)	RVS	712 (49.69)	179 (64.39)	533 (46.15)	<0.0001
	SVS	721 (50.31)	99 (35.61)	622 (53.85)	
Age (mean (SD))		37.486 (15.331)	26.450 (10.712)	40.143 (15.090)	<0.0001
Sex (%)	Female	593 (41.38)	115 (41.37)	478 (41.39)	1
	Male	840 (58.62)	163 (58.63)	677 (58.61)	
Diagnose (%)	Asymptomatic infection	859 (59.94)	155 (55.76)	704 (60.95)	0.1287
	Mild	574 (40.06)	123 (44.24)	451 (39.05)	
VS (mean (SD))		10.426 (2.613)	9.849 (2.522)	10.565 (2.616)	<0.0001
CT3minORF (mean (SD))		22.252 (5.024)	21.183 (3.611)	23.639 (6.146)	<0.0001
CT3minN (mean (SD))		20.827 (5.535)	19.523 (3.686)	22.503 (6.906)	<0.0001
Alcohol (%)	No	1325 (92.46)	238 (85.61)	1087 (94.11)	<0.0001
	Yes	108 (7.54)	40 (14.39)	68 (5.89)	
Smoke (%)	No	1309 (91.35)	233 (83.81)	1076 (93.16)	<0.0001
	Yes	124 (8.65)	45 (16.19)	79 (6.84)	
DM (%)	No	1420 (99.09)	278 (100.00)	1142 (98.87)	0.1542
	Yes	13 (0.91)	0 (0.00)	13 (1.13)	
HP (%)	No	1400 (97.70)	277 (99.64)	1123 (97.23)	0.029
	Yes	33 (2.30)	1 (0.36)	32 (2.77)	
Fever (%)	No	910 (63.50)	172 (61.87)	738 (63.90)	0.5752
	Yes	523 (36.50)	106 (38.13)	417 (36.10)	
Vaccine (%)	No	81 (14.94)	0 (0.00)	81 (19.10)	<0.0001
	Yes	461 (85.06)	118 (100.00)	343 (80.90)	
WBC (mean (SD))		5.772 (1.978)	6.004 (1.799)	5.718 (2.015)	0.1798
Hb (mean (SD))		145.259 (17.716)	143.160 (18.638)	145.742 (17.483)	0.1764
Plt (mean (SD))		214.049 (57.141)	218.340 (49.948)	213.063 (58.676)	0.3918
Neu (mean (SD))		4.094 (8.516)	5.879 (19.207)	3.680 (1.863)	0.016
Lym (mean (SD))		1.381 (0.604)	1.337 (0.621)	1.392 (0.600)	0.3995
CRP (mean (SD))		11.456 (14.445)	12.004 (14.425)	11.329 (14.462)	0.6648

n, number of patients; VS group, Viral Shedding group; RVS, Rapid Viral Shedding; SVS, Slow Viral Shedding; CT3minORF, Lowest ORF1ab-CT values for Day1-Day3; CT3minN, Lowest N-CT values for Day1-Day3; DM, diabetes mellitus; WBC, White Blood Cell; HP, Hypertension; Plt, Platelets; Neu, neutrophils; Lym, Lymphocyte cell; CRP, C-reactive protein.

Unpaired t test (two sides), was used in two group measurement data. ANOVA test was used in in multi groups' measurement data. Fisher's exact test was used in enumeration data.

Supplementary Table 2. Comparison between SVS and RVS groups of Omicron BA.4/5 or BF.7 patients.

Characteristics	Level	SVS	RVS	p
n		721	712	
Group (%)	BA.4/5	99 (13.73)	179 (25.14)	<0.0001
	BF.7	622 (86.27)	533 (74.86)	
Age (mean (SD))		40.786 (15.072)	34.145 (14.870)	<0.0001
Sex (%)	Female	311 (43.13)	282 (39.61)	0.1929
	Male	410 (56.87)	430 (60.39)	
Diagnose (%)	Asymptomatic infection	409 (56.73)	450 (63.20)	0.0144
	Mild	312 (43.27)	262 (36.80)	
VS (mean (SD))		12.423 (1.524)	8.403 (1.805)	<0.0001
CT3minORF (mean (SD))		20.623 (3.579)	23.266 (5.510)	<0.0001
CT3minN (mean (SD))		18.899 (3.762)	22.019 (6.097)	<0.0001
Alcohol (%)	No	673 (93.34)	652 (91.57)	0.2425
	Yes	48 (6.66)	60 (8.43)	
Smoke (%)	No	667 (92.51)	642 (90.17)	0.1382
	Yes	54 (7.49)	70 (9.83)	
DM (%)	No	710 (98.47)	710 (99.72)	0.0274
	Yes	11 (1.53)	2 (0.28)	
HP (%)	No	700 (97.09)	700 (98.31)	0.1699
	Yes	21 (2.91)	12 (1.69)	
Fever (%)	No	463 (64.22)	447 (62.78)	0.6104
	Yes	258 (35.78)	265 (37.22)	
Vaccine (%)	No	48 (17.27)	33 (12.50)	0.1513
	Yes	230 (82.73)	231 (87.50)	
WBC (mean (SD))		5.704 (2.151)	5.843 (1.782)	0.4048
Hb (mean (SD))		145.714 (17.537)	144.783 (17.922)	0.5324
Plt (mean (SD))		212.838 (59.500)	215.318 (54.642)	0.6059
Neu (mean (SD))		4.364 (11.790)	3.810 (1.669)	0.4388
Lym (mean (SD))		1.387 (0.596)	1.375 (0.613)	0.8061
CRP (mean (SD))		11.768 (14.852)	11.135 (14.033)	0.6036
