# Supplementary figures and images for: A robust web-based tool to predict viral shedding in patients with Omicron SARS-CoV-2 variants
Source: ERJ Open Res. 2024 May 20;10(3):00939-2023. doi: 10.1183/23120541.00939-2023 (PMC11111115; doi:10.1183/23120541.00939-2023)

# Supplementary Figure 1

A

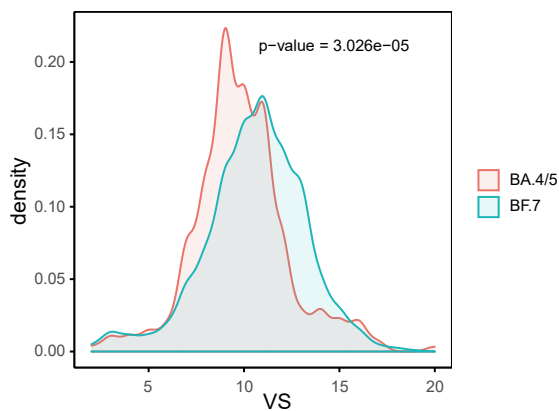

B

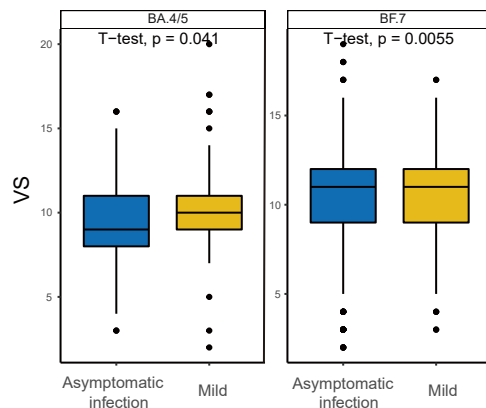

C

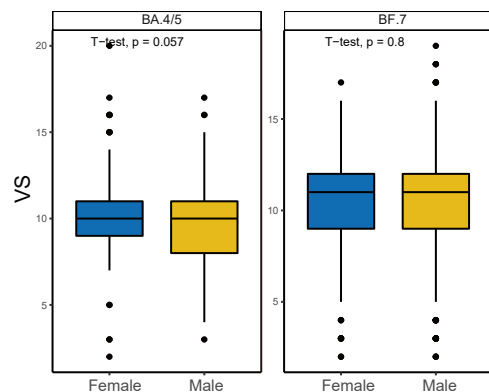

D

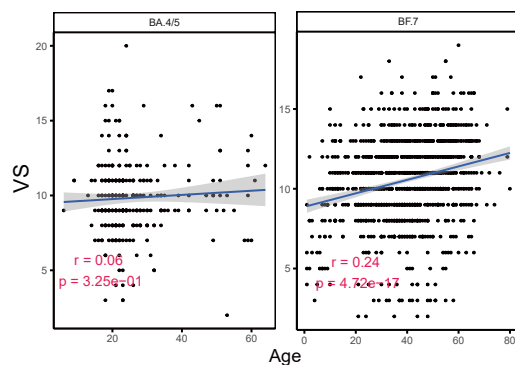

E

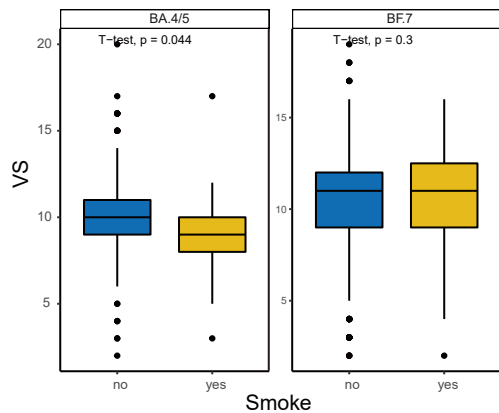

F

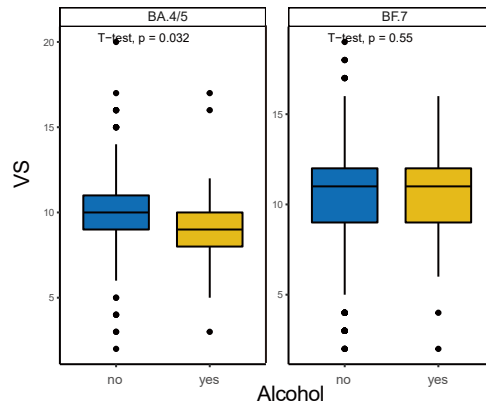

# Supplementary Figure 2

A

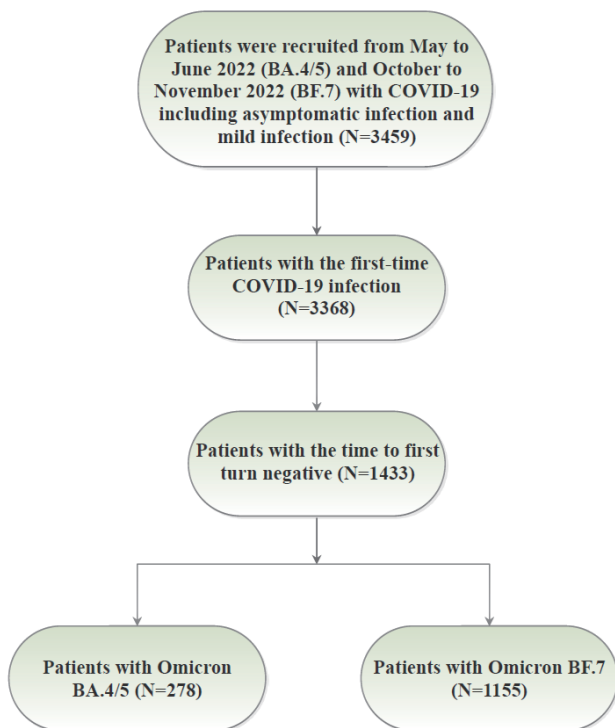

B

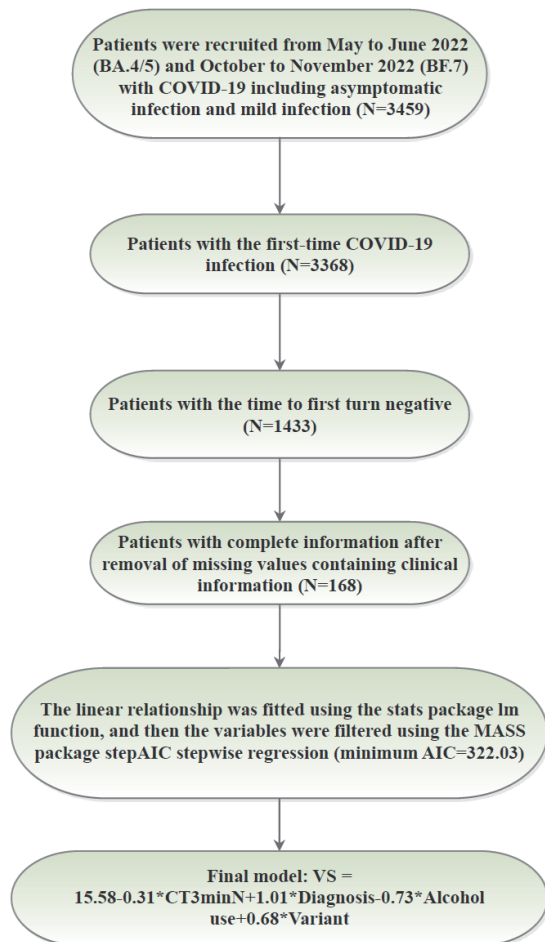

# Supplementary Figure 3

A

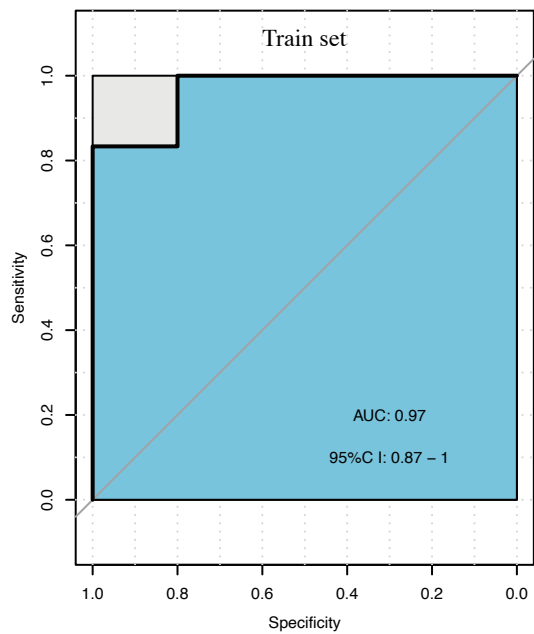

B

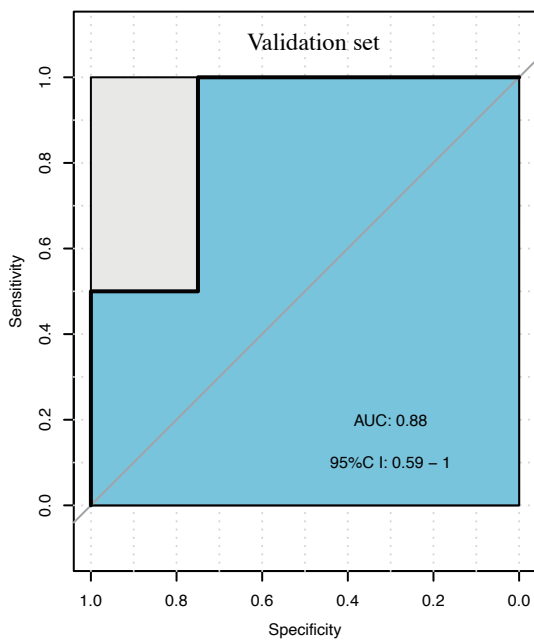

Supplement: Supplementary file 3 [file 00939-2023.SUPPLEMENT3.pdf]
